# Supplementary material for: Patterns of Intron Gain and Loss in Fungi
Source: PLoS Biol. 2004 Nov 30;2(12):e422. doi: 10.1371/journal.pbio.0020422 (PMC532390; doi:10.1371/journal.pbio.0020422)
Supplement: Table S1 — Also available at http://genes.mit.edu/NielsenEtAl/. (4.3 MB ZIP). [file pbio.0020422.st001.zip › NielsenEtAl/html/911.html]

AN5049.1.NCU09368.1.MG04623.1.FG01162.1


```
 CLUSTAL W (1.82) Multiple Sequence Alignments - Introns Inserted


Sequence 1: NCU09368.1	543 aa
Sequence 2: MG04623.1	561 aa
Sequence 3: FG01162.1	587 aa
Sequence 4: AN5049.1	558 aa
Alignment Length: 609 aa
Number Identitical Residues: 186 aa
Alignment Score (without introns) 9837


MG04623.1 	MTSPNG--SSSSYK--RQSSFLLLQGEDHPFPVAPKGNSALASLIRSRG--SFTSSTHPT
NCU09368.1	MASPN---YAHSYPGGRSGPFLCLQ---EPQPPAPKGHSALASLIRNRPGISLPNPLNPF
FG01162.1 	MSSPNTPSKHGSHGQFRRPSFLSND---DSHNPLGTRSGHLQSLIRSRSQQSMQSLSSSL
AN5049.1  	MTRNMKLSRCQCGVVSRRRGAVAEKRSQSDISGANNQPSSQYCLIDPRMASSGRRTRPQF
          	*:     :   .    *    :  . ..       .  .   .**  *   *        

MG04623.1 	GPH--------DEESGAYGDDVDADEHNRARDHERRLSAILNGPHMRSMRLIGKNNPRYQ
NCU09368.1	RDE--------EEALAGLGVERRLVDDESPRRDERRMSAVLNGPHMRSMRLIGNSNPRYR
FG01162.1 	PAHSGAFWMGDDEETGLLRHDN--DELGRLLADERRLTQLLHGPQNRSAKLIGRSNPRYR
AN5049.1  	SVPSSP---SDNESPIRPRRPSLEPQVDILSTRGQYLSSLIRRRSFSSSQSLYRPDGYID
          	   :..   ..:*            :        : :: ::.     * : : . :    

MG04623.1 	WEK-----YWKTEEELSQMKR-----------SI~REYYERTNYLIQQYLYIDRLLDSSL
NCU09368.1	WER-----YWKTEDELATMKK-----------SI2REYYERCNYLVQQYLYIDKLLDSSL
FG01162.1 	WEQ-----YWRPEEELASMPK-----------HL2RDYYERTNDLIQQYLYIDALLDSAI
AN5049.1  	TERDDTGSFRHRVEGPATVRARELIGERRPPYNW2RQLYERNNNLISQYVYIDRLLDSSL
          	 *:..:.:: :  :  : :   .  ..  ..    *: *** * *:.**:*** ****::

MG04623.1 	PHDLLNEYN-YMPASAFRG-VEIPETISEE----HNSHGTPSTESLRGSSPGGTVSGSGN
NCU09368.1	PHDLLNEYN-DLPPWAFRGGLEAPVAT------------PPTSGAAAEPAP---------
FG01162.1 	PHDLLNEYQAELDASAFRP-VDVPDTINEEPSISTSETSPLNTSTPASITPGSYGAISVK
AN5049.1  	PRRLIVEYQ---QNRAVES----------------------AKRTNAGAHEGGYGSFENL
          	*: *: **:      *..                        . :      ..  : .  

MG04623.1 	GTPAGNRK-VKRTPQDIYRHTETTPLLMLPEDDDGIDEVHDPDEETPLVQ----KHDIPW
NCU09368.1	-TFSKTRK-VKRTPKDIYRPTETTPLFCG--DGNAVDDE-DSDQVASQET----RPEIPW
FG01162.1 	HSNSSTKLPATRTPQDIFRSSENLPLLQHQDHDDDEEHEVQPSQPANKDDDNGPRPLLPW
AN5049.1  	ADDEIPKK-IKRTPRNLYRIPSESSLLLP----QTTDGEASPLEDVTPKG----------
          	      :   .***::::* ..  .*:      :  :   .. : .              

MG04623.1 	IEDDNVDSTAPIVTLAIYINFAANAILLAGKIAVIVTVPSVSVLASLVDAALDFLSTAIV
NCU09368.1	LEDDDVDSSASIVTLAIYINFAANAILLVGKLAVVLTVPSVSVLASLVDAILDFLSTAIV
FG01162.1 	LEDAEIDSDDPIVTLAIWVNFIANAVLLAGKLVVIMSVPSMSVLASLVDAVLDFLSTVIV
AN5049.1  	--HDFVDSGARIVTVAIYVNFVANVILLAAKIAALLMTNSVSVLASLVDGALDFLSTTIV
          	  .  :**   ***:**::** **.:**..*:..:: . *:********. ******.**

MG04623.1 	WVTTWTIT--QQDQYRYPVGRRR~LEPIGVLVFSIIMCTAFCQVALEAITRLMSGDHEVI
NCU09368.1	WITTWLIS--RQDQYRYPIGRRR~LEPIGVLVFSVIMITSFAQVALEAIQRLMSNDREVI
FG01162.1 	WVTTRLISSSQSDQHSYPVGRRK2LEPLGVLVFSIIMITSFCQVGLECIQRLMSPEHPIL
AN5049.1  	WITTALIR--RQDRYEYPISRRR~LEPLSVLVFAVVMVTSFFQVAITSAGRLISSEHAVI
          	*:**  *   :.*:: **:.**: ***:.****:::* *:* **.: .  **:* :: ::

MG04623.1 	SL2GIPAIAIMFSTIVIKGMCWLWCRLVKNSSVQAL2AADALTDVIFNAGSIAFPIA1GS
NCU09368.1	EL2GVPAIAIMLSTVVIKGMCWLWCRLIKNSSVQAL2ASDASTDVIFNAGSIAFPLI1GY
FG01162.1 	EL~GVPAIAIMVSTIVIKGACWIWCRVVKNSSVRAL2AEDAKTDVIFNTGSILFPII1GF
AN5049.1  	QL~SVPSAAIMGSTVVVKFLCWFWCRLINNSSVQAL~AQDAMTDVVFNFFSIVFPLV1GG
          	.* .:*: *** **:*:*  **:***:::****:** * ** ***:**  ** **:  * 

MG04623.1 	FLKIWWLDALGGLLLSLVVIINWSSNAGEHIKNLAGFSATADQRNIL~LYLTMRFAKTIK
NCU09368.1	YCQIWWLDALGGLLLSLVVIFNWSQTSGEHIRHLTGFSATADQRNIL1LYLTMRFAKTIK
FG01162.1 	YGRVWWLDAFGGLFLSLVVIFNWSQTSAHHVRNLSGFSAQPDERNLL~LYLTMRFATAIR
AN5049.1  	FTNIWFLDPLGGLILSIYIIWNWSRTASEHIRHLTGAAASREDVSIL~LYMTMRFSRSIL
          	: .:*:**.:***:**: :* *** .:..*:::*:* :*  :: .:* **:****: :* 

MG04623.1 	QIQGLQAYHSGDKLTVEVDIVLDASTSLKDSHDLA2ESLQYVIESVPIVDRAFVHTDYAT
NCU09368.1	QIQGLQAYHSGDKLNVEVDIVLDASTSLKDSHDLA2ESLQYVIESVPIVDRAFVHVDYAS
FG01162.1 	QIQNLRAYHAGDKLFVEVDIVLSAATPLKDSHDLS~EVLTYFLESVPIVDRAFVHVDYTS
AN5049.1  	KIQNLRAYYAGDLLNVEVDIVLPDNSSLRDSHDLG~ESLQYMLESVPTVERAFVHMDYDP
          	:**.*:**::** * *******   :.*:*****. * * *.:**** *:***** ** .

MG04623.1 	YNLPTHMQQQS-----
NCU09368.1	YNLPTHMEQQHNI---
FG01162.1 	YNAPTHMLKGSAAKPK
AN5049.1  	WNIPSHMNQQEA----
          	:* *:** :
```
